# Comparison of next-generation sequencing samples using compression-based distances and its application to phylogenetic reconstruction

**DOI:** 10.1186/1756-0500-7-320

**Published:** 2014-05-29

**Authors:** Ngoc Hieu Tran, Xin Chen

**Affiliations:** 1 School of Physical and Mathematical Sciences, Nanyang Technological University, Singapore, Singapore

**Keywords:** Alignment-free sequence comparison, Sequence distance, Sequence compression, Next-generation sequencing

## Abstract

**Background:**

Enormous volumes of short read data from next-generation sequencing (NGS) technologies have posed new challenges to the area of genomic sequence comparison. The multiple sequence alignment approach is hardly applicable to NGS data due to the challenging problem of short read assembly. Thus alignment-free methods are needed for the comparison of NGS samples of short reads.

**Results:**

Recently several *k*-mer based distance measures such as *CVTree*, $d_{2}^{S}$, and *co-phylog* have been proposed or enhanced to address this problem. However, how to choose an optimal *k* value for those distance measures is not trivial since it may depend on different aspects of the sequence data. In this paper, we considered an alternative parameter-free approach: compression-based distance measures. These measures have shown good performance for the comparison of long genomic sequences, but they have not yet been tested on NGS short reads. Hence, we performed extensive validation in this study and showed that the compression-based distances are highly consistent with those distances obtained from the *k*-mer based methods, from the multiple sequence alignment approach, and from existing benchmarks in the literature. Moreover, as the compression-based distance measures are parameter-free, no parameter optimization is required and these measures still perform consistently well on multiple types of sequence data, for different kinds of species and taxonomy levels.

**Conclusions:**

The compression-based distance measures are assembly-free, alignment-free, parameter-free, and thus represent useful tools for the comparison of long genomic sequences as well as the comparison of NGS samples of short reads.

## Background

Recent advances in next-generation sequencing (NGS) technologies have produced massive amounts of short read data, bringing up promising opportunities in many biomedical research areas such as RNA-seq, ChIP-seq, *de novo* whole genome sequencing, metagenome sequencing, etc [1]. The short read data also poses new challenges to the field of genomic sequence analysis, including the problem of sequence comparison. Sequence distance measures are often applied to compare long genomic sequences such as 16S rRNA sequences, mtDNA sequences, gene encoding sequences, or even whole genome sequences [2-4]. The obtained distances are then used for sequence clustering and classification, for phylogenetic tree reconstruction, for inference of the evolution and relationship of species, etc.

However, with the development of NGS technologies, a new type of sequence data emerges: NGS short reads are orders of magnitudes shorter than long genomic sequences while being generated at unprecedented high throughput. Hence, it is highly desirable to go beyond the comparison of long genomic sequences to develop new methods for the comparison of NGS samples of millions of short reads [5].

The multiple sequence alignment (MSA) approach is hardly applicable to large data sets of NGS short reads due to its prohibitive computational cost and the challenging problem of short read assembly, especially for species without any reference genomes (*de novo* assembly). Alignment-free methods [4] could overcome these limitations of the alignment-based approach. They are assembly-free and scalable to large data sets. Most existing alignment-free methods use *k*-mers (*k*-tuples or *k*-words) as sequence signatures to measure sequence distances [6-10]. Markov models were also proposed for DNA sequence comparison [11], and could be incorporated with *k*-mer distributions to achieve more accurate distances [12,13].

Recently several studies have further refined existing techniques or developed new ones for better applications to NGS short read data. In particular, the following three measures have shown impressive performance on both NGS short reads and long genomic sequences: *CVTree*[6,7], $d_{2}^{S}$[14,15] and *co-phylog*[10]. For a given *k*, *CVTree* and $d_{2}^{S}$ measure the distance between two NGS samples (or two DNA sequences) based on the normalized *k*-mer frequencies. *co-phylog*, on the other hand, computes the distance from the average nucleotide substitution rate in the observed *k*-mers.

The *k*-mer based measures, however, depend considerably on the parameter *k*. A non-optimal choice of *k* could lead to a dramatically worse result in some cases. In general, a larger value of *k* allows the measures to use more parameters to better capture the full characteristics of the input sequences (or NGS samples). However, there might not be sufficient data available for an accurate estimation of a large number of parameters. Moreover, the optimal *k* value may depend on the types of sequence data, the species of interest, and even the taxonomy level. When the measures are applied to NGS data, we need to consider even more factors such as the NGS platform, the sequencing depth, the read length, etc. Thus, how to choose an optimal *k* is a very challenging task.

In this paper, we consider an alternative parameter-free approach: compression-based distance measures [16-18]. Roughly speaking, data compression is aimed at reducing as much redundant information in the given data as possible. Hence, if two NGS samples share similar patterns, compressing them together should be more efficient, that is, should use less storage space, than compressing them separately. The distance between the two NGS samples can then be calculated based on the sizes of the reduced storage space. Readers are referred to [16,17,19,20] for more formal theory about sequence complexity, compression and distance metrics. Compression-based distances have been successfully applied to many clustering and classification problems with data of various types, including DNA sequences, texts and languages, time series, images, sound, video [16-19,21,22]. However, they have not been tested on NGS short reads yet.

In this study we demonstrate that the compression-based distance measures can be successfully applied not only to long genomic sequences (including 16S rRNA, mtDNA, and whole genome sequences) but also to NGS samples of short reads. Extensive validation was conducted to assess the accuracy of the compression-based and *k*-mer based distances on four data sets: 29 mammalian mtDNA sequences, 29 *Escherichia/Shigella* genomes, 70 *Gammaproteobacteria* genomes, and 39 mammalian gut metagenomic samples. The data sets include various types of genomic sequences, in silico and real NGS short reads, different species and taxonomy levels. The validation results show that the compression-based distances are highly consistent with those distances obtained from the *k*-mer based methods, from the MSA approach, and from existing benchmarks in the literature. Our results also show that the *k*-mer based distance measures depend critically on the choice of *k*, and the optimal *k* varies across different data sets. In contrast, the compression-based distance measures are parameter-free and thus perform consistently well on all data sets without any optimization of parameters. The details are presented in the following sections.

## Methods

### Compression-based distance measures

Let *x* and *y* denote the two sequences (or NGS samples) to be compared and *xy* denote their concatenation. Let *C*(*x*) denote the size (that is, the number of bytes) of *x* after being compressed by a sequence compression tool. Data compression is aimed at reducing as much redundant information in the given data as possible. Hence, if *x* and *y* share similar patterns, compressing them together should use less storage space than compressing them separately, that is, *C*(*xy*) ≤ *C*(*x*) + *C*(*y*). Specifically, if *x* and *y* are identical, one could expect that *C*(*xy*) ≃ *C*(*x*) = *C*(*y*). On the other hand, if *x* and *y* share no information, one could expect that *C*(*xy*) ≃ *C*(*x*) + *C*(*y*). These observations suggest that one could measure the similarity/dissimilarity between *x* and *y* based on their compressed sizes.

In particular, the following distance measure called compression-based dissimilarity measure (CDM) was proposed in [18]:

(1) $$d^{\text{CDM}}(x,y)=\frac{C(\text{xy})}{C(x)+C(y)}.$$

This *d*^CDM^ distance ranges from $\frac{1}{2}$ (when *x* and *y* are identical) to 1 (when *x* and *y* share no information).

A more mathematically precise distance was proposed in [16] using the notation of conditional compression:

(2) $$d(x,y)=\frac{C(x|y)+C(y|x)}{C(\text{xy})}.$$

Here *C*(*x*|*y*) denotes the compressed size of sequence *x* conditioning on sequence *y*. *C*(*x*|*y*) ≃ 0 indicates that *x* and *y* are identical, while *C*(*x*|*y*) ≃ *C*(*x*) indicates that *x* and *y* share no information and thus they are expected to be independent sequences. This distance ranges from 0 to 1 and satisfies the triangle inequality [16].

The authors further refined the distance *d* and proposed the following in [17] which they referred to as normalized compression-based distance (NCD):

(3) $$d^{\text{NCD}}(x,y)=\frac{\text{max}\{C(x|y),C(y|x)\}}{\text{max}\{C(x),C(y)\}}.$$

Moreover, they have shown that the *d*^NCD^ distance is a proper metric, satisfying the non-negativity, identity, symmetry, and triangle inequality axioms. Readers are referred to [16,17,19,20] for more formal theory about sequence complexity, compression and distance metrics.

In principle, any sequence compression tool can be used to compute the above distances. More efficient compression should lead to more accurate distance estimates, but may require longer compression time. In this study, we used the tool *GenCompress*[23] since it can perform conditional compression on *x*|*y*. We used the same compression tool for both long genomic sequences and NGS short reads to ensure a consistent and fair comparison. When applying *GenCompress* to an NGS sample, we first concatenated all short reads together to form a single sequence and then compressed it. Our experiment results show that the compression-based distances are quite robust against different orders of concatenating the short reads.

### *k*-mer based distance measures

In this study, we considered three *k*-mer based distance measures: *CVTree*[6,7], $d_{2}^{S}$[14,15] and *co-phylog*[10]. Given two DNA sequences (or two NGS samples), *CVTree* measures the correlation distance between their composition vectors, where each composition vector is the collection of the normalized frequencies of *k*-mers. The $d_{2}^{S}$ distance is an NGS-extension of the *D*_2_, $D_{2}^{*}$, and $d_{2}^{S}$ statistics which were proposed in [8,9] for the comparison of long genomic sequences. The main difference between $d_{2}^{S}$ and *CVTree* lies in the normalization of the frequencies of *k*-mers. The *co-phylog* distance is also based on *k*-mers but not in the frequency context. It measures the distance as the average nucleotide substitution rate in the observed *k*-mers of the two sequences (or samples).

We used the implementations provided by the authors. The tools *CVTree* and $d_{2}^{S}$ have options to input the parameter *k*. For *CVTree*, we tried *k* from 3 to 32 as allowed by the tool. For $d_{2}^{S}$, we were not able to run it for *k* > 9 due to some “segmentation fault” error, which seems to be a problem of handling dynamic memory in the tool. There is no input option for *co-phylog*, thus we simply used its default settings.

### Data sets

We examined the above six alignment-free distance measures *d*^NCD^, *d*, *d*^CDM^, *CVTree*, $d_{2}^{S}$, and *co-phylog* on both NGS short reads and long genomic sequences (including 16S rRNA, mtDNA, and whole genome sequences). The sequences and short reads were retrieved or simulated from four data sets: 29 mammalian mtDNA sequences [13,17,19,24], 29 *Escherichia/Shigella* genomes [10], 70 *Gammaproteobacteria* genomes [10], and 39 metagenomic mammalian gut samples [14,25].

The tool *MetaSim*[26] was used to simulate short reads from genomic sequences. It offers four error models: 454, Sanger, Empirical (Illumina), and Exact, corresponding to three different NGS platforms and the non-error case, respectively. We set the read length to be 100 and used default settings for other parameters. Short reads were simulated at four sampling depths: 1×, 5×, 10×, and 30×.

For applications to real NGS data sets, we recommend that the sequencing coverage should be 5× or higher. In order to ensure a fair comparison, it is also desirable that the samples are produced from the same NGS platforms, with similar experimental conditions, sequencing coverage, read lengths, etc. Strictly identical numbers of reads or identical read lengths, however, are not necessary. For example, the real NGS data set of 39 metagenomic samples analyzed in our study was produced from the 454 FLX platform, with a total of 2,163,286 reads (an average of 55,469 ± 28,724 (standard deviation, SD) reads per sample, 261 ± 83 nucleotides per read) [25].

### Accuracy assessment

To assess the accuracy of the alignment-free distances, we compared them with those obtained from the MSA approach if applicable and with existing benchmarks in the literature. The tool Clustal Omega [27] was used to perform MSA and then the tool *dnadist* in the package PHYLIP [28] was used to calculate the distance matrix from the MSA.

Following [10,14], we used distance correlation, tree symmetric difference, and parsimony score to measure the accuracy of the alignment-free distances. In particular, we computed the correlation between each alignment-free distance and the MSA/benchmark distance to evaluate their consistency. The correlation between two distance matrices was calculated as follows. We first converted each matrix into a single vector by concatenating all of its rows side by side and then calculated the Pearson correlation between the two vectors.

We also assessed the alignment-free distances by examining their corresponding phylogenetic trees. For each distance matrix, the tool *neighbor* in the package PHYLIP was used to construct a phylogenetic tree using the neighbor joining method [29]. Subsequently, the tool *treedist* in the package PHYLIP was used to calculate the symmetric difference [30] between the resulting tree from each alignment-free distance and the corresponding MSA/benchmark tree. Each internal node in a phylogenetic tree corresponds to a subset of clustered leaf nodes. Given two phylogenetic trees with the same set of leaf nodes, the symmetric difference between them is the number of internal nodes that are present in one tree but not in the other.

Finally, to assess the clustering and classification ability of the alignment-free distances, we used the parsimony score to measure how different a clustering tree is from the true classification (using tools *TreeClimber*[31], *mothur*[32]). The parsimony score of a clustering tree is calculated by the tool *TreeClimber* as follows. First, the parsimony score is set to 0 and the leaf nodes are labeled according to their groups in the true classification. The algorithm traverses from the leaf nodes to the root and determine the labels of the internal nodes. The labels of each internal node depend on the labels of its two immediate child nodes. If they share common labels then these common labels are assigned to the internal node. If the two child nodes share no label, a penalty of 1 is added to the parsimony score and the internal node is assigned with the union of the label sets of its two child nodes. If a clustering tree is perfect, its parsimony score is equal to the number of groups in the true classification minus one. The higher the parsimony score is, the more different the clustering tree is from the true classification.

The tool TreeGraph 2 [33] was used to plot phylogenetic trees.

## Results and discussion

### Alignment-free comparison of mammalian mtDNA sequences or their NGS short reads reconfirms the hypothesis (Rodents, (Ferungulates, Primates))

One of the key advantages of the alignment-free distance measures over the alignment-based approach is their scalability to large data sets of whole genome sequences or NGS short reads. However, in this section we first want to assess their accuracy on a small, but very well-studied data set of 29 mammalian mtDNA sequences. This data set has been widely used for validation in existing literatures and hence reliable benchmarks are available [13,17,19,24].

#### Performance on mtDNA sequences

First, we applied the six alignment-free distance measures *d*^NCD^, *d*, *d*^CDM^, *CVTree*, $d_{2}^{S}$, *co-phylog* to the mtDNA sequences and compared the results with those obtained from the MSA method and from existing benchmarks. Additional file 1: Table S1 shows that both compression-based distances *d*^NCD^, *d*, *d*^CDM^ and *k*-mer based distances *CVTree*, $d_{2}^{S}$ (for optimal choices of *k*) are in good agreement with the MSA distance. The $d_{2}^{S}$ distance has the highest correlation with the MSA distance, whereas the *CVTree* tree has the smallest symmetric difference from the MSA tree. The compression-based distances, leading by *d*^CDM^, performed slightly worse. The *co-phylog* measure, however, failed for this data set. One possible explanation is that *co-phylog* may be only suitable for closely related species, as the authors mentioned in [10]. We also noted that the *CVTree* and $d_{2}^{S}$ distances varied remarkably with respect to *k* (Additional file 1: Table S2). For instance, the smallest symmetric difference between the *CVTree* tree and the MSA tree is 6 (*k* = 10), but the largest is up to 48 (*k* = 16, 17). Similarly, the highest correlation between the $d_{2}^{S}$ distance and the MSA distance is 0.88 (*k*=8), but the lowest is down to 0.41 (*k*=3).

The MSA tree (Additional file 2: Figure S1a) is highly consistent with existing benchmarks in the literature [13,17,19,24]. In particular, the MSA tree is nearly identical to those reported in [13,19], except for two minor differences in the branches of dog, cat and the branches of non-murid rodents (fat dormouse, squirrel, guinea pig). Additional file 2: Figures S1a and S1b show that the main difference between the MSA tree and the *d*^CDM^ tree also lies in the group Rodents. In addition, the *d*^CDM^ tree indicates that pig is closer to cow and sheep than to other species in the group Ferungulates. The branches of dog, cat in the *d*^CDM^ tree are slightly different from those in the MSA tree, but consistent with previously reported trees in [13,19]. The trees reported by *CVTree* and $d_{2}^{S}$ (Additional file 2: Figures S1c and S1d, respectively) also show different results for the group Rodents. The phylogeny of the group Rodents is actually still a controversial question, as mentioned in previous studies [13,19]. The position of the cluster of dog, cat, and seals in the group Ferungulates reported by *CVTree* is not consistent with the other trees and the benchmarks. Overall, all four trees support the hypothesis of (Rodents, (Ferungulates, Primates)), as suggested in [13,17,19,24], and have identical phylogeny of the group Primates. The main differences among them include the phylogeny of the group Rodents, the positions of pig and hippo, and the subtree of dog, cat and seals in the group Ferungulates.

#### Performance on NGS short reads

Next, we ask if similar results could be obtained from the comparison of NGS samples of short reads. We used the tool *MetaSim*[26] to simulate short reads from the mtDNA sequences with four error models 454, Exact, Empirical (Illumina), Sanger, and four different sampling depths 1×, 5×, 10×, and 30×. The read length was set at 100 bp. We used *k* = 10 for the *CVTree* distance and *k* = 8 for the $d_{2}^{S}$ distance as suggested by their optimal performance on the mtDNA sequences in the previous section. Since the MSA method is difficult to apply to NGS short reads, we still kept the MSA distance and tree obtained from the mtDNA sequences as benchmark. At the 1× sampling depth, we found that the alignment-free results were considerably different from the MSA benchmark due to the low coverage. However, at the 5× sampling depth, all five measures *d*^NCD^, *d*, *d*^CDM^, *CVTree*, and $d_{2}^{S}$ produced comparably accurate results as when they were applied to the mtDNA sequences. Further increasing the sampling depth to 10× and 30× did not significantly improve the accuracy of the distances.

Table 1 summarizes the results for the 5× sampling depth, similar results for 1×, 10×, and 30× can be found in Additional file 1: Table S2. We highlighted in boldface both the best and the second best of the tree symmetric difference and the distance correlation for each error model because they are usually not very different. As shown in Table 1, the $d_{2}^{S}$ distance achieved the highest correlation with the MSA distance, followed by the *CVTree* distance.

**Table 1:** Comparison of the alignment-free distances and the MSA distance for NGS short reads of the mtDNA sequences

	***d***^**NCD**^	***d***	***d***^**CDM**^	***CVTree* (*k*** **=10)**	$d_{2}^{S}(k=8)$
454	14	16	**10**	**10**	**6**
Exact	**8**	**8**	**8**	**8**	**8**
Empirical	**6**	8	**4**	8	12
Sanger	**10**	14	**8**	14	**8**
454	0.68	0.66	0.68	**0.75**	**0.88**
Exact	0.69	0.68	0.68	**0.71**	**0.88**
Empirical	**0.69**	**0.69**	0.66	**0.69**	**0.81**
Sanger	0.67	0.67	0.65	**0.74**	**0.87**

The short reads were simulated from the mtDNA sequences using four error models 454, Exact, Empirical, and Sanger of the tool *MetaSim* at 5 × sampling depth. The two smallest tree symmetric differences and the two highest distance correlation coefficients for each error model are highlighted in boldface. Similar results for 1 ×, 10 ×, and 30 × sampling depths can be found in Additional file 1: Table S2.

**Figure 1:**
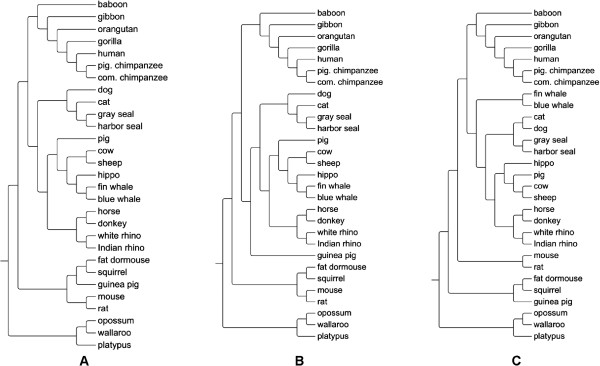
**Phylogenetic trees reconstructed from NGS short reads of 29 mtDNA sequences using: (A) *d***^***CDM***^**, (B) *CVTree *(*k *= 10), (C) **$d_{2}^{S}(k=8)$**.** The short reads were simulated from the tool *MetaSim* using the Empirical model and 5× sampling depth. The group of three species platypus, opossum, and wallaroo was used as the outgroup to root the tree.

In terms of the symmetric difference from the MSA tree, the *d*^CDM^ distance performed consistently well for all four error models, followed by the $d_{2}^{S}$ and *d*^NCD^ distances. Figure 1 shows the phylogenetic trees reconstructed from the *d*^CDM^, *CVTree*, and $d_{2}^{S}$ distances for the NGS short reads simulated using the Empirical (Illumina) error model. The *d*^CDM^ and *CVTree* trees are almost identical to the MSA tree (Additional file 2: Figure S1a) and existing benchmarks in the literature [13,17,19,24], supporting the hypothesis (Rodents, (Ferungulates, Primates)).

$$d_{2}^{S}(k=8)$$

The $d_{2}^{S}$ tree, however, has more inconsistent branches in the group Ferungulates, although it has the highest correlation with the MSA distance. Last but not least, we also noted that the alignment-free results obtained from the simulated NGS short reads were consistent with their corresponding counterparts obtained from the mtDNA sequences in the previous section, especially for the non-error (Exact) model (Table 2).

**Table 2:** Comparison of the phylogenetic trees reconstructed from the mtDNA sequences and from their NGS short reads

	***d***^**NCD**^	***d***	***d***^**CDM**^	***CVTree* (*k *** **= 10)**	$d_{2}^{S}(k=8)$
454	10	14	**8**	**8**	**4**
Exact	**2**	**0**	4	**2**	**2**
Empirical	**8**	**6**	**6**	**6**	10
Sanger	12	**10**	**8**	**10**	**8**

The short reads were simulated from the mtDNA sequences using four error models 454, Exact, Empirical, and Sanger of the tool *MetaSim* at 5 × sampling depth. The two smallest tree symmetric differences for each error model are highlighted in boldface. Similar results for 1 ×, 10 ×, and 30 × sampling depths can be found in Additional file 1: Table S2.

When applying *GenCompress* to an NGS sample, we concatenated all short reads of the sample to form a single sequence and then compressed it. Hence, it is also important to examine if the compression-based distances are robust against different ways of concatenation. We repeated the experiment with the Empirical (Illumina) samples for 10 different runs in each of which the reads from each sample were concatenated in a random order. Additional file 1: Table S8a shows that the compression-based distances obtained from those runs are highly consistent with each other. We also compared those distances with the MSA distance and the performance results (Additional file 1: Table S8b) are similar to those reported earlier in Table 1.

In summary, we have shown that all five alignment-free distance measures *d*^NCD^, *d*, *d*^CDM^, *CVTree*, and $d_{2}^{S}$ can be successfully applied to both mtDNA sequences and their NGS short reads. The distances obtained from the NGS short reads were consistent with those obtained from the mtDNA sequences, and they were all in good agreement with the MSA distance as well as with existing benchmarks in the literature. The compression-based measures *d*^NCD^, *d*and *d*^CDM^ produced comparably accurate distances as those optimal results obtained from the *k*-mer based measures *CVTree* and $d_{2}^{S}$. The compression-based distances were also shown to be quite robust against different concatenations of short reads.

The *CVTree* and $d_{2}^{S}$ distances varied remarkably with respect to *k*, and the optimal *k* was selected according to the MSA benchmark. This may pose a challenging problem when there is no benchmark available for validation. In contrast, the compression-based measures are parameter-free and hence no optimization of any parameter is required.

### Phylogeny of closely related *Escherichia/Shigella* genomes

In this section we assess the accuracy of the alignment-free distances on a data set of 29 *Escherichia/Shigella* genomes. Two main differences between this data set and the previous one are: (i) it consists of whole genome sequences and (ii) the species are closely related bacteria in the genus *Escherichia* and the genus *Shigella*. This data set has been studied previously in [10,34] and the authors have shown that the *co-phylog* distance was highly consistent with the MSA distance in terms of both tree symmetric difference and distance correlation. Hence, to avoid the time-consuming MSA, we used the *co-phylog* distance as benchmark.

#### Performance on whole genome sequences

We first applied the five measures *d*^NCD^, *d*, *d*^CDM^, *CVTree*, and $d_{2}^{S}$ to the whole genome sequences and compared the results with the benchmark obtained from the *co-phylog* measure. Table 3 shows that the *d*^CDM^ distance performed the best in terms of both tree symmetric difference and distance correlation. The results of *d*^NCD^ and *d*are also better than the best results of *CVTree* and $d_{2}^{S}$, especially with the remarkably high correlation with the benchmark *co-phylog* distance. The $d_{2}^{S}$ distance failed for this data set and its correlation with the benchmark *co-phylog* distance is much lower than that of the other measures. The phylogenetic trees produced by the *CVTree* distance are inconsistent for different values of *k*: it is not clear whether the genus *Shigella* violates the monophyleticity of the genus *Escherichia* (*k* = 15) or the monophyleticity of the *E.coli* strains (*k* = 9, 21) (Additional file 2: Figure S2). This was also mentioned previously in [10].

**Table 3:** **Comparison of the alignment-free distances and the benchmark *co-phylog *** **distance for 29 *Escherichia/Shigella *** **genomes**

	***d***^**NCD**^	***d***	***d***^**CDM**^	***CVTree *** **(*k *** **= 9)**	***CVTree *** **(*k *** **= 15)**	***CVTree *** **(*k *** **= 21)**	$d_{2}^{S}(k=8)$
Symmetric difference	16	**14**	**12**	20	20	16	24
Distance correlation	**0.97**	0.95	**0.99**	0.80	0.79	0.80	0.20

The two smallest tree symmetric differences and the two highest correlation coefficients are highlighted in boldface.

#### Performance on NGS short reads

**Figure 2:**
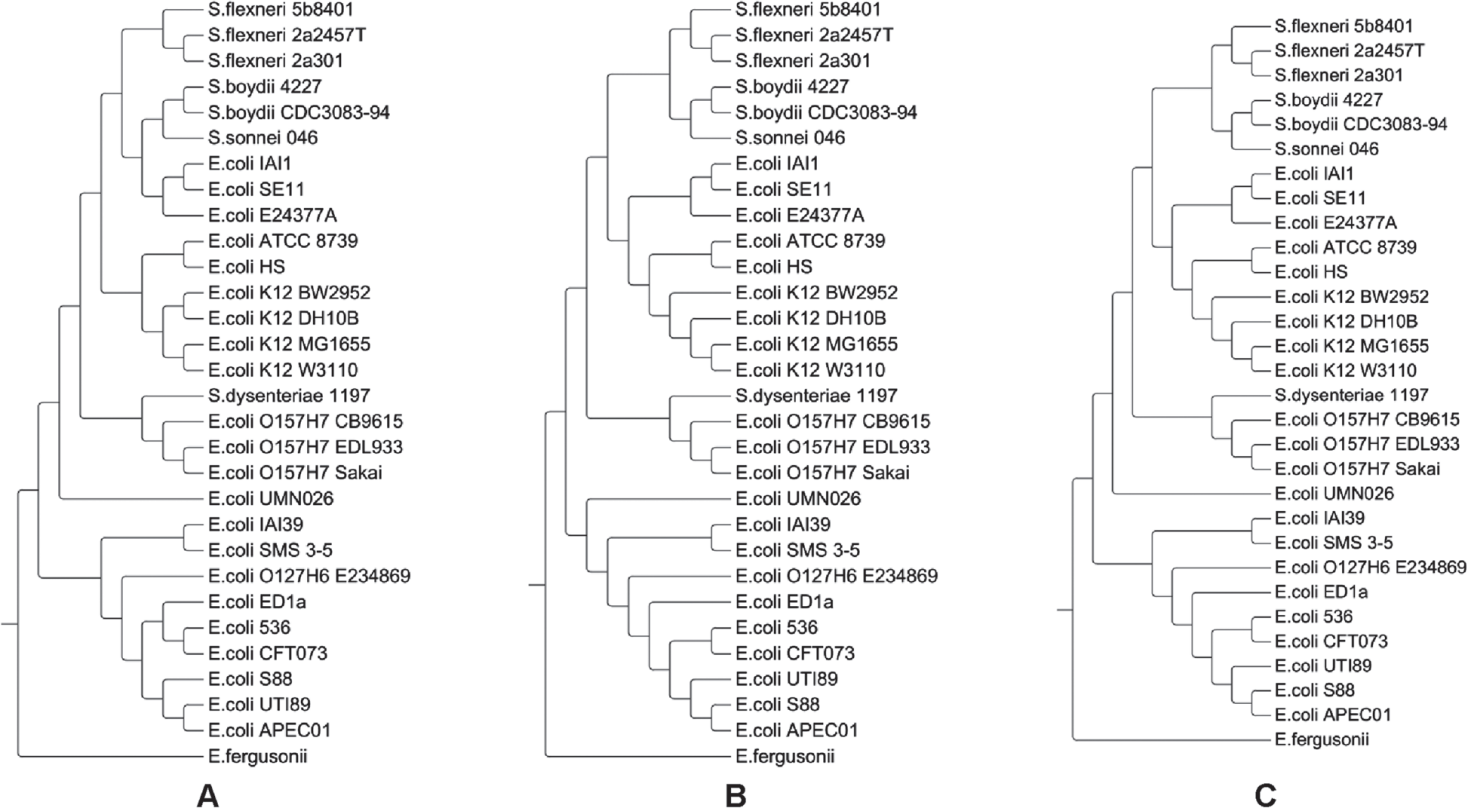
**Phylogenetic trees reconstructed from 29 *Escherichia/Shigella * genomes using (A) *co-phylog*, (B)*d***^***CDM***^**, and from NGS short reads using (C) *d***^***CDM***^**.** The short reads were simulated from the tool *MetaSim* using the Exact model and 1× sampling depth. *Escherichia Fergusonii* was used as the outgroup to root the tree.

Next, we tested the measures on the data sets of NGS short reads which were simulated from the whole genome sequences. We used *MetaSim* with four error models and different sampling depths as described earlier. Interestingly, even at the lowest 1× sampling depth, we already obtained accurate results from the three compression-based distances *d*^NCD^, *d*, and *d*^CDM^. Figure 2 shows the case of the *d*^CDM^ distance in which the phylogenetic tree reconstructed from the NGS short reads (Exact model) is almost identical to the tree reconstructed from the whole genome sequences. Moreover, both trees are very similar to the benchmark *co-phylog* tree. The main difference is that in the benchmark *co-phylog* tree the group of *S.boydii* and *S.sonnei* is clustered with *E.coli* first, whereas in the *d*^CDM^ trees this group is clustered with *S.flexneri* first. Table 4 clearly shows that the compression-based distances *d*^NCD^, *d*, and *d*^CDM^ outperformed the *CVTree* and $d_{2}^{S}$ distances for all NGS data sets, in terms of both tree symmetric difference and distance correlation.

**Table 4:** **Comparison of the alignment-free distances and the benchmark *co-phylog *** **distance for NGS short reads of 29 *Escherichia/Shigella *** **genomes**

	***d***^**NCD**^	***d***	***d***^**CDM**^	***CVTree *** **(*k *** **= 9)**	***CVTree *** **(*k *** **= 15)**	***CVTree *** **(*k *** **= 21)**	$d_{2}^{S}(k=8)$
454	16	**14**	**12**	22	22	54	50
Exact	**12**	**10**	**10**	22	24	36	50
Empirical	16	**12**	**14**	24	18	42	54
Sanger	**12**	**10**	**10**	28	24	40	50
454	**0.96**	**0.96**	**0.97**	0.74	0.87	0.31	0.04
Exact	**0.97**	**0.97**	**0.98**	0.82	0.82	0.61	0.05
Empirical	**0.96**	**0.96**	**0.96**	0.77	0.85	0.38	0.01
Sanger	**0.97**	**0.97**	**0.97**	0.78	0.86	0.36	0.04

The short reads were simulated from the *Escherichia/Shigella* genomes using four error models 454, Exact, Empirical, and Sanger of the tool *MetaSim* at 1 × sampling depth. The two smallest tree symmetric differences and the two highest correlation coefficients for each error model are highlighted in boldface. Similar results for 5 × sampling depth can be found in Additional file 1: Table S3.

We also noted that while the results of the compression-based distances for the whole genome sequences (Table 3) and for the NGS short reads (Table 4) were comparable, the performance of the *CVTree* and $d_{2}^{S}$ distances became worse when they were applied to the NGS short reads. Similar results for the NGS data sets obtained from the 5× sampling depth can be found in Additional file 1: Table S3.

In summary, the compression-based distances *d*^NCD^, *d*, and *d*^CDM^ were consistent with the benchmark *co-phylog* distance on both types of data, whole genome sequences and NGS short reads of 29 *Escherichia/Shigella* bacteria. They outperformed the two *k*-mer based distances *CVTree* and $d_{2}^{S}$, which either failed or produced inconsistent results for different values of *k*. The results in this section further emphasize the wide applicability and the consistency of the compression-based distances. They represent useful measures for accurate comparison of different types of long genomic sequences and NGS short read data, for both mammalian and bacteria species.

### Classification of 70 genomes in the class *Gammaproteobacteria* into their correct orders

The previous section has focused on closely related bacteria at the genus level. We next applied the MSA and the alignment-free distance measures to a larger data set at a higher taxonomy level. In particular, the data set consists of 70 genomes that were randomly chosen from 15 orders of the class *Gammaproteobacteria* (Additional file 1: Table S4). As the number of genomes is large and they come from different groups, it is interesting to ask if the distance measures can cluster and classify those genomes into their correct orders. We used the parsimony score to measure the difference between a clustering tree and the true classification [31,32].

As the number of groups is 15, the optimal parsimony score is 14. The higher the parsimony score is, the more different the clustering tree is from the true classification. This data set has been studied previously in [10] and the authors found that the *co-phylog* distance did not perform well because the bacteria of interest are not closely related.

#### Performance on 16S rRNA sequences

As it is challenging to perform MSA of 70 whole genome sequences, we applied the MSA method to 16S rRNA sequences of those 70 genomes to obtain the benchmark distance and clustering tree (Additional file 2: Figure S3, parsimony score = 18).

**Figure 3:**
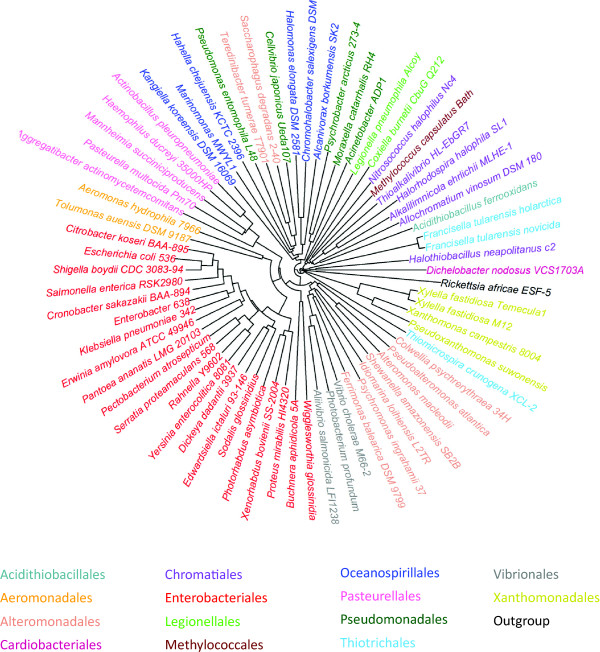
**Clustering tree reconstructed from 16S rRNA sequences of 70 *Gammaproteobacteria *genomes using the *d***^***NCD ***^**distance.** Those 70 genomes belong to 15 orders which are indicated by different colors in the figure.

We then applied all six alignment-free distance measures *d*^NCD^, *d*, *d*^CDM^, *CVTree*, $d_{2}^{S}$, and *co-phylog* to the 16S rRNA sequences. Table 5 shows that the alignment-free distances are highly correlated with the MSA distance and they all have similar parsimony scores (17-18), except for the *co-phylog* distance. Overall, the *d*^NCD^ distance performed slightly better than the others in terms of parsimony score, tree symmetric difference, and distance correlation. Its clustering tree in Figure 3 shows that the genomes of six orders *Aeromonadales*, *Enterobacteriales*, *Legionellales*, *Pasteurellales*, *Vibrionales*, and *Xanthomonadales* are all correctly classified into their groups. Most of the genomes in the remaining orders are also well clustered.

**Table 5:** **Comparison of the alignment-free distances and the benchmark MSA distance for 70 *Gammaproteobacteria *** **genomes**

		***d***^**NCD**^	***d***	***d***^**CDM**^	***CVTree***	$d_{2}^{S}$	***co-phylog***
	parsimony score	17	**18**	**18**	**17**	**18**	25
16s rRNA sequences	tree symmetric difference	**50**	**52**	**52**	**50**	62	108
	distance correlation	**0.93**	0.90	**0.93**	**0.92**	**0.92**	0.65
	parsimony score	**22**	**22**	**21**	**21**	31	26
Genome sequences	tree symmetric difference	80	**78**	**76**	84	110	110
	distance correlation	0.47	0.46	0.47	**0.67**	**0.50**	0.45
	parsimony score	**21**	**19**	23	24	32	28
NGS short reads	tree symmetric difference	90	**70**	**84**	88	114	116
	distance correlation	**0.60**	0.58	0.53	**0.63**	0.48	0.42

The NGS short reads were simulated from the whole genome sequences using the Exact model of *MetaSim* at 1 × sampling depth. The two smallest parsimony scores, the two smallest tree symmetric differences and the two highest correlation coefficients are highlighted in boldface. For *CVTree*, we used *k* = 7 for the 16S rRNA data set and *k* = 12 for the whole genome and NGS data sets. For *d* 2*S*, we used *k* = 6 for the 16S rRNA data set and *k* = 8 for the whole genome and NGS data sets.

#### Performance on whole genome sequences and their NGS short reads

Then, we applied the alignment-free distance measures to the 70 whole genome sequences and their simulated NGS short reads.

The clustering results obtained from the NGS short reads are comparable to those obtained from the whole genome sequences, and both are worse than those obtained from the 16S rRNA sequences (Table 5). Since this experiment was conducted at a high taxonomy level and the species were selected from different orders of the class *Gammaproteobacteria*, one could expect that the 16S rRNA sequences should be more reliable for the classification than the whole genome sequences and their short reads. It can also be seen from Table 5 that the four distances *d*^NCD^, *d*, *d*^CDM^, and *CVTree* performed slightly better than the other two distances, $d_{2}^{S}$ and *co-phylog*, especially for the whole genome sequences and NGS short reads. Last but not least, we noted that the optimal *k* of *CVTree* and $d_{2}^{S}$ for the whole genome sequences were different from those for the 16S rRNA sequences (Additional file 1: Table S5). The optimal *k* was selected to optimize the parsimony score of the clustering trees. This will not be possible if we have no prior knowledge about the true classification, which is usually the case in real applications.

### Classification of metagenomic samples from mammalian gut reveals the diet and gut physiology of the host species

In this section we consider a real metagenomic data set that includes NGS short reads of 39 fecal samples from 33 mammalian host species. The host species can be classified into four groups according to their diet and gut physiology: foregut-fermenting herbivores (13 samples), hindgut-fermenting herbivores (8 samples), carnivores (7 samples), and omnivores (11 samples) (Additional file 1: Table S6). This data set has been studied previously in [14,25]. In [14] the authors applied the *CVTree* and $d_{2}^{S}$ distances to these 39 metagenomic samples and found that the sequence signatures (that is, the *k*-mers) of the samples were strongly associated with the diet and gut physiology of the host species. Hence we want to test if the compression-based distance measures *d*^NCD^, *d*and *d*^CDM^ can also reveal any interesting results from this metagenomic data set.

#### Performance on the sub-data set with 11 omnivore samples excluded

Following [14,25], we first excluded 11 omnivore samples due to their complicated microbial compositions. The remaining 28 samples belong to three groups: foregut-fermenting herbivores, hindgut-fermenting herbivores, and carnivores. Since there is no benchmark tree for this clustering problem, we only used the parsimony score to evaluate the clustering trees. The optimal parsimony score is 2 as there are only three groups in the true classification.

Table 6 shows that the parsimony scores of the *CVTree* and $d_{2}^{S}$ distances for the optimal *k* are better than those of the compression-based distances *d*^NCD^, *d*, and *d*^CDM^. We also noted that the parsimony score of the *CVTree* distance varied considerably (up to 11), while that of the $d_{2}^{S}$ distance was more stable (Additional file 1: Table S7).

**Table 6:** Parsimony score for the classification of 39 metagenomic samples using the alignment-free distances

	***d***^**NCD**^	***d***	***d***^**CDM**^	***CVTree***	$d_{2}^{S}$
Sub-data set	6	7	**5**	**3**	**3**
(omnivore samples excluded)					
Full data set	**9**	12	12	**10**	**10**

For *CVTree*, we used *k* = 6 for the full data set and *k* = 4 for the sub-data set in which the omnivore samples were excluded. For *d*${}_{\text{2}}^{\text{S}}$, we used *k* = 7 for the full data set and *k* = 5 for the sub-data set. The two smallest parsimony scores for each data set are highlighted in boldface.

The optimal tree obtained from the $d_{2}^{S}$ distance (*k* = 5, parsimony score = 3) is shown in Additional file 2: Figure S6. Only two samples Rock Hyrax 1 and 2 were wrongly classified to the group of hindgut-fermenting herbivores. Although the optimal tree obtained from the *CVTree* distance (*k* = 4) also has the same parsimony score of 3, it seems to have a serious mistake when classifying the two carnivores Polar Bear and Lion to the groups of herbivores (Additional file 2: Figure S7). The clustering tree obtained from the *d*^CDM^ distance (parsimony score = 5) is shown in Additional file 2: Figure S8. It correctly distinguished carnivores from herbivores. However, it wrongly classified Rock Hyraxes, Colobus and Visayam Warty Pig to the group of hindgut-fermenting herbivores.

#### Performance on the full data set

Then, we added back the 11 omnivore samples and repeated the experiment with the full data set. As there are now four groups in the true classification, the optimal parsimony score is 3.

We found that the best parsimony score was obtained from the *d*^NCD^ distance, followed by *CVTree* (*k* = 6) and $d_{2}^{S}$ (*k* = 7) (Table 6). It should be noted that the optimal *k* of the *CVTree* and $d_{2}^{S}$ distances for the sub-data set and for the full data set are different (Table 6, Additional file 1: Table S7).

**Figure 4:**
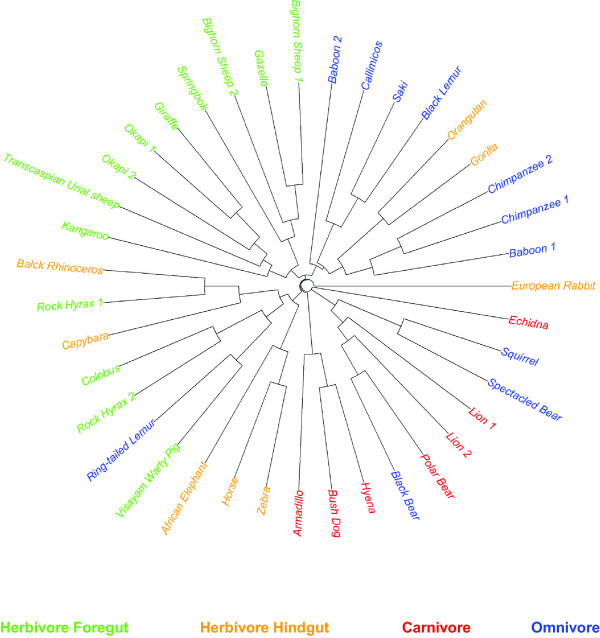
**Clustering tree reconstructed from 39 metagenomic samples using the *d***^***NCD ***^**distance.** The host species’ colors indicate their diet and gut physiology: foregut-fermenting herbivores (green), hindgut-fermenting herbivores (yellow), carnivores (red) and omnivores (blue).

The clustering tree of the *d*^NCD^ distance is shown in Figure 4. The samples from foregut-fermenting herbivores were well clustered together, except for Rock Hyraxes, Colobus, and Visayam Warty Pig, which were classified to the group of hindgut-fermenting herbivores. This is similar to the earlier observation when the 11 omnivore samples were excluded. Figure 4 also shows that the carnivore samples were grouped together. The omnivore samples, however, were scattered throughout the groups of herbivores and carnivores. This indicates the diversity of the gut microbial communities of omnivores, as mentioned previously in [14,25]. Another important observation from Figure 4 is that the primates samples, including Baboon 1 and 2, Chimpanzee 1 and 2, Orangutan, Gorilla, Callimicos, Saki, Black Lemur, were clustered together into one group. This may suggest that those primates share common features in their gut microbial environments. Finally, it can be seen that two samples of the same host species were often clustered close to each other such as Chimpanzee 1 and 2, Lion 1 and 2, Okapi 1 and 2, Bighorn Sheep 1 and 2, supporting the accuracy of the classification and the *d*^NCD^ distance.

The results obtained in this section have demonstrated another application of the alignment-free measures of sequence distance: comparison and classification of metagenomic samples of NGS short reads. This task is of critical importance for the understanding of microbial communities. Both *k*-mer based and compression-based distance measures have revealed interesting results about the microbial communities of mammalian gut from their metagenomic samples. In particular, the information contained in the samples was found to be strongly associated with the diet and gut physiology of herbivores, carnivores, and omnivores. This agrees well with previous studies in [14,25]. Moreover, our results obtained from the compression-based distance measures also discovered a strong similarity between the gut microbial communities of the primates. This interesting finding has not been observed in previous studies.

## Conclusions

In this paper we studied the application of compression-based distance measures for the problem of sequence comparison, with a special focus on NGS short read data. Their key advantages are assembly-free, alignment-free, and parameter-free. We conducted extensive validation on various types of sequence data: NGS short reads, 16S rRNA sequences, mtDNA sequences, and whole genome sequences. The sequence data was obtained from several mammalian and bacteria genomes at different taxonomy levels, as well as from microbial metagenomic samples. The results show that the compression-based distance measures produced comparably accurate results as the *k*-mer based methods, and both were in good agreement with the alignment-based approach and with existing benchmarks in the literature.

The *k*-mer based distance measures, however, may produce inconsistent results depending on the parameter *k*, the type of sequence data, or the species under consideration. For example, the *co-phylog* measure was not applicable to species with far evolutionary distances from each other (data set of 29 mammalian, data set of 70 *Gammaproteobacteria*, data set of 39 metagenomic samples). The $d_{2}^{S}$ measure failed for the data set of 29 closely related *Escherichia/Shigella* bacteria. The *CVTree* measure produced inconsistent results for the data set of 29 *Escherichia/Shigella* bacteria. The compression-based measures, although not always producing the best distances, performed consistently well across all data sets in the study without any optimization required. We believe such a consistent performance is due to their parameter-free feature. On the other hand, choosing an optimal parameter *k* for each data set is of critical importance for using the *k*-mer based methods. This task would become daunting when there is no benchmark (e.g., true phylogenetic trees or true classifications) available to guide the analysis and the selection of *k*.

One possible drawback when using the compression-based distance measures is the running time. Obviously, compressing a DNA sequence (or an NGS sample) takes longer time than counting its *k*-mers. Moreover, the compression-based methods need to perform pairwise compression of the input sequences, whereas the *k*-mer methods only need to calculate one frequency vector for each input sequence. However, in general one may also need to test a wide range of *k* to find the optimal results when using the *k*-mer methods.

As an example, for the data set of 39 metagenomic samples in our study, the running time of the *CVTree* measure was ∼1-7 minutes for each *k* = 2, 3, …, 10, and ∼10-60 minutes for each *k* = 11, 12, …, 20. Thus, a test covering all values of *k* = 2, 3, …, 10 only took less than 20 minutes, but if all the values of *k* = 11, 12, …, 20 were included, the running time increased up to ∼8 hours.

The running time when using GenCompress to calculate the compression-based measures for this data set was ∼25 hours, about 3 times longer than that of *CVTree*. Compression tools developed specifically for NGS short reads such as *BEETL*[35] and *SCALCE*[36] can be applied to reduce the running time. We also expect that such NGS compression tools should be more efficient and hence provide more accurate distances. Our future research will focus on reducing the running time and studying the effects of different compression tools.

In this study we have demonstrated the accuracy and the consistency of the compression-based distance measures on both NGS short reads and long genomic sequences. Those findings underscore the advantages of the compression-based distance measures, suggesting that these measures represent useful tools for the alignment-free sequence comparison. An implementation of the compression-based distance measures is provided in the attached Perl scripts (Additional file 3).

## Competing interests

The authors declare that they have no competing interests.

## Authors’ contributions

XC designed the project. NHT performed the analysis and wrote the paper. Both authors have read and approved the manuscript.

## Supplementary Material

Additional file 1 **Table S1.** Comparison of alignment-free distances and the benchmark MSA distance for 29 mtDNA sequences. **Table S2.** Comparison of alignment-free distances and the benchmark MSA distance for 29 mtDNA sequences and their short reads. **Table S3.** Comparison of alignment-free distances and the benchmark *co-phylog* distance for 29 *Escherichia/Shigella* genomes and their short reads. **Table S4.** The list of 70 genomes in the class *Gammaproteobacteria*, their orders, and their accession numbers. **Table S5.** Comparison of alignment-free distances and the benchmark MSA distance for 70 *Gammaproteobacteria* genomes and their short reads. **Table S6.** The list of 39 metagenomic samples and their host species’ diet and gut physiology. **Table S7.** The parsimony score of the clustering trees for 39 metagenomic samples. **Table S8.** Performance of the compression-based distances on 10 different random concatenations of the NGS short reads simulated from the mtDNA sequences using the Empirical (Illumina) model. Click here for file

Additional file 2 **Figure S1.** Phylogenetic trees reconstructed from 29 mtDNA sequences using: (a) MSA, (b) *d*^CDM^, (c) *CVTree* (*k* = 10), (d) $d_{2}^{S}(k=8)$. **Figure S2.** Phylogenetic trees reconstructed from 29 *Escherichia/Shigella* genomes using: (a) *co-phylog*, (b) *CVTree* (*k* = 9), (c) *CVTree* (*k* = 15), (d) *CVTree* (*k* = 21). **Figure S3.** Clustering tree reconstructed from 16S rRNA sequences of 70 *Gammaproteobacteria* genomes using the MSA distance. **Figure S4.** Clustering tree reconstructed from 16S rRNA sequences of 70 *Gammaproteobacteria* genomes using the distance *CVTree* (*k* = 7). **Figure S5.** Clustering tree reconstructed from 16S rRNA sequences of 70 *Gammaproteobacteria* genomes using the distance $d_{2}^{S}$ (*k* = 6). **Figure S6.** Clustering tree reconstructed from the metagenomic samples (omnivore samples excluded) using the distance $d_{2}^{S}(k=5)$. **Figure S7.** Clustering tree reconstructed from the metagenomic samples (omnivore samples excluded) using the distance *CVTree* (*k* = 4). **Figure S8.** Clustering tree reconstructed from the metagenomic samples (omnivore samples excluded) using the distance *d*^CDM^. Click here for file

Additional file 3 Perl scripts and the example data set of 29 mtDNA sequences. Click here for file
