# Supplementary material for: Comparison of next-generation sequencing samples using compression-based distances and its application to phylogenetic reconstruction
Source: BMC Res Notes. 2014 May 29;7:320. doi: 10.1186/1756-0500-7-320 (PMC4057587; doi:10.1186/1756-0500-7-320)
Supplement: Additional file 2 — Figure S1. Phylogenetic trees reconstructed from 29 mtDNA sequences using: (a) MSA, (b) dCDM, (c) CVTree (k = 10), (d) d2S(k=8). Figure S2. Phylogenetic trees reconstructed from 29 Escherichia/Shigella genomes using: (a) co-phylog, (b) CVTree (k = 9), (c) CVTree (k = 15), (d) CVTree (k = 21). Figure S3. Clustering tree reconstructed from 16S rRNA sequences of 70 Gammaproteobacteria genomes using the MSA distance. Figure S4. Clustering tree reconstructed from 16S rRNA sequences of 70 Gammaproteobacteria genomes using the distance CVTree (k = 7). Figure S5. Clustering tree reconstructed from 16S rRNA sequences of 70 Gammaproteobacteria genomes using the distance d2S (k = 6). Figure S6. Clustering tree reconstructed from the metagenomic samples (omnivore samples excluded) using the distance d2S(k=5). Figure S7. Clustering tree reconstructed from the metagenomic samples (omnivore samples excluded) using the distance CVTree (k = 4). Figure S8. Clustering tree reconstructed from the metagenomic samples (omnivore samples excluded) using the distance dCDM. [file 1756-0500-7-320-S2.pdf]

Figure S1

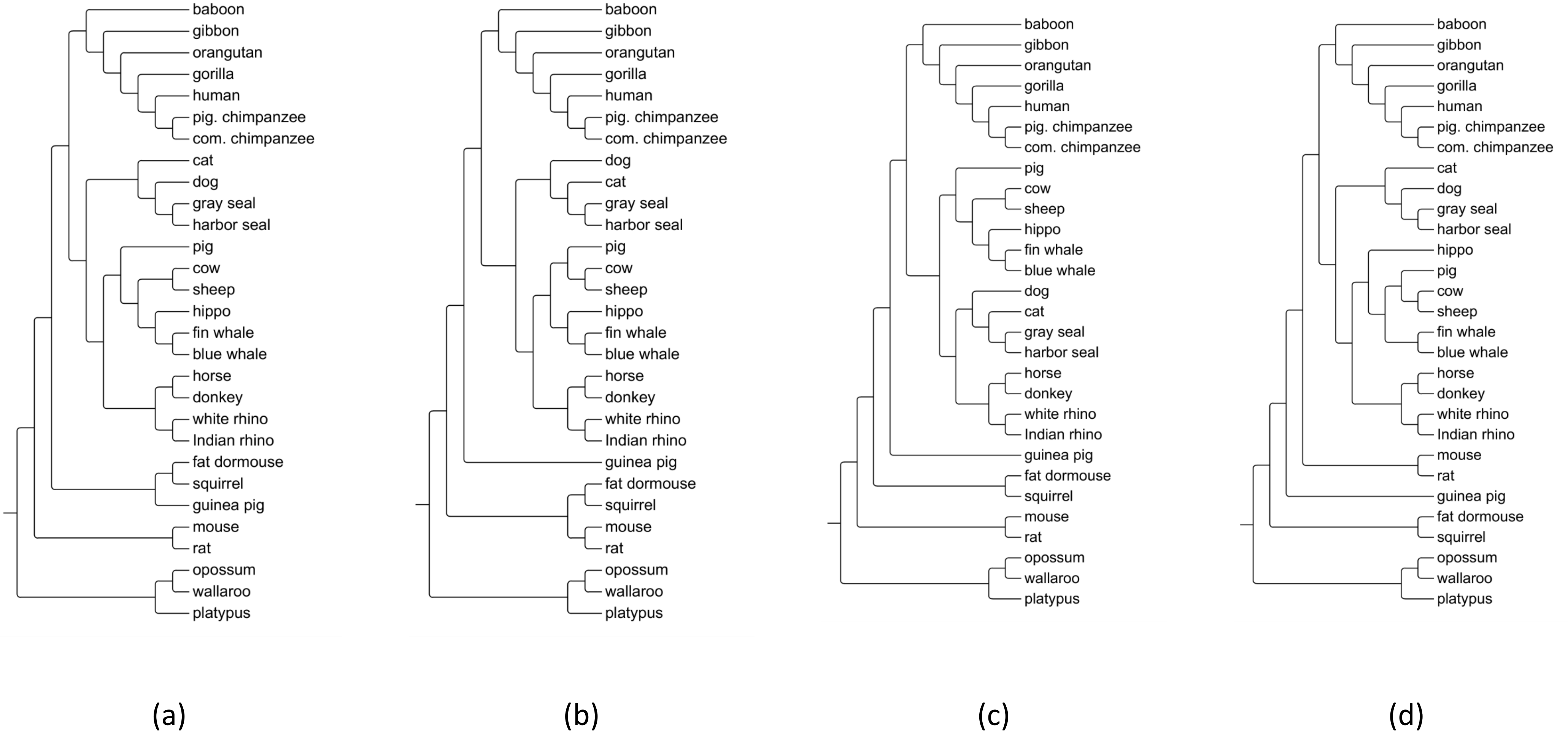

baboon
gibbon
orangutan
gorilla
human
pig. chimpanzee
com. chimpanzee
cat
dog
gray seal
harbor seal
pig
cow
sheep
hippo
fin whale
blue whale
horse
donkey
white rhino
Indian rhino
fat dormouse
squirrel
guinea pig
mouse
rat
opossum
wallaroo
platypus
(a)
baboon
gibbon
orangutan
gorilla
human
pig. chimpanzee
com. chimpanzee
dog
cat
gray seal
harbor seal
pig
cow
sheep
hippo
fin whale
blue whale
horse
donkey
white rhino
Indian rhino
guinea pig
fat dormouse
squirrel
mouse
rat
opossum
wallaroo
platypus
(b)
baboon
gibbon
orangutan
gorilla
human
pig. chimpanzee
com. chimpanzee
pig
cow
sheep
hippo
fin whale
blue whale
dog
cat
gray seal
harbor seal
horse
donkey
white rhino
Indian rhino
guinea pig
fat dormouse
squirrel
mouse
rat
opossum
wallaroo
platypus
(c)
baboon
gibbon
orangutan
gorilla
human
pig. chimpanzee
com. chimpanzee
cat
dog
gray seal
harbor seal
hippo
pig
cow
sheep
fin whale
blue whale
horse
donkey
white rhino
Indian rhino
mouse
rat
guinea pig
fat dormouse
squirrel
opossum
wallaroo
platypus
(d)

Figure S2

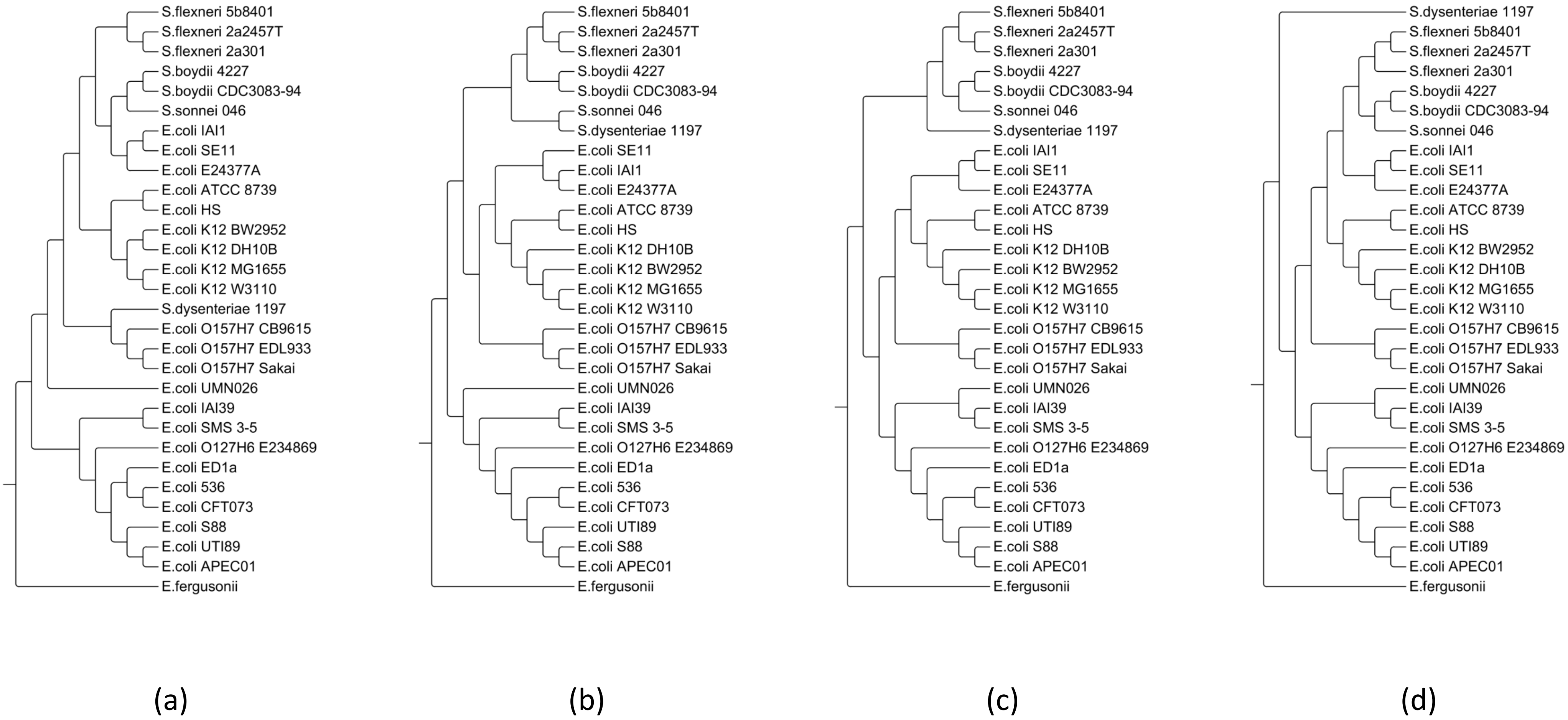
S.flexneri 5b8401
S.flexneri 2a2457T
S.flexneri 2a301
S.boydii 4227
S.boydii CDC3083-94
S.sonnei 046
E.coli IAI1
E.coli SE11
E.coli E24377A
E.coli ATCC 8739
E.coli HS
E.coli K12 BW2952
E.coli K12 DH10B
E.coli K12 MG1655
E.coli K12 W3110
S.dysenteriae 1197
E.coli O157H7 CB9615
E.coli O157H7 EDL933
E.coli O157H7 Sakai
E.coli UMN026
E.coli IAI39
E.coli SMS 3-5
E.coli O127H6 E234869
E.coli ED1a
E.coli 536
E.coli CFT073
E.coli S88
E.coli UTI89
E.coli APEC01
E.fergusonii
(a)
S.flexneri 5b8401
S.flexneri 2a2457T
S.flexneri 2a301
S.boydii 4227
S.boydii CDC3083-94
S.sonnei 046
S.dysenteriae 1197
E.coli SE11
E.coli IAI1
E.coli E24377A
E.coli ATCC 8739
E.coli HS
E.coli K12 DH10B
E.coli K12 BW2952
E.coli K12 MG1655
E.coli K12 W3110
E.coli O157H7 CB9615
E.coli O157H7 EDL933
E.coli O157H7 Sakai
E.coli UMN026
E.coli IAI39
E.coli SMS 3-5
E.coli O127H6 E234869
E.coli ED1a
E.coli 536
E.coli CFT073
E.coli UTI89
E.coli S88
E.coli APEC01
E.fergusonii
(b)
S.flexneri 5b8401
S.flexneri 2a2457T
S.flexneri 2a301
S.boydii 4227
S.boydii CDC3083-94
S.sonnei 046
S.dysenteriae 1197
E.coli IAI1
E.coli SE11
E.coli E24377A
E.coli ATCC 8739
E.coli HS
E.coli K12 DH10B
E.coli K12 BW2952
E.coli K12 MG1655
E.coli K12 W3110
E.coli O157H7 CB9615
E.coli O157H7 EDL933
E.coli O157H7 Sakai
E.coli UMN026
E.coli IAI39
E.coli SMS 3-5
E.coli O127H6 E234869
E.coli ED1a
E.coli 536
E.coli CFT073
E.coli UTI89
E.coli S88
E.coli APEC01
E.fergusonii
(c)
S.dysenteriae 1197
S.flexneri 5b8401
S.flexneri 2a2457T
S.flexneri 2a301
S.boydii 4227
S.boydii CDC3083-94
S.sonnei 046
E.coli IAI1
E.coli SE11
E.coli E24377A
E.coli ATCC 8739
E.coli HS
E.coli K12 BW2952
E.coli K12 DH10B
E.coli K12 MG1655
E.coli K12 W3110
E.coli O157H7 CB9615
E.coli O157H7 EDL933
E.coli O157H7 Sakai
E.coli UMN026
E.coli IAI39
E.coli SMS 3-5
E.coli O127H6 E234869
E.coli ED1a
E.coli 536
E.coli CFT073
E.coli S88
E.coli UTI89
E.coli APEC01
E.fergusonii
(d)

Figure S3

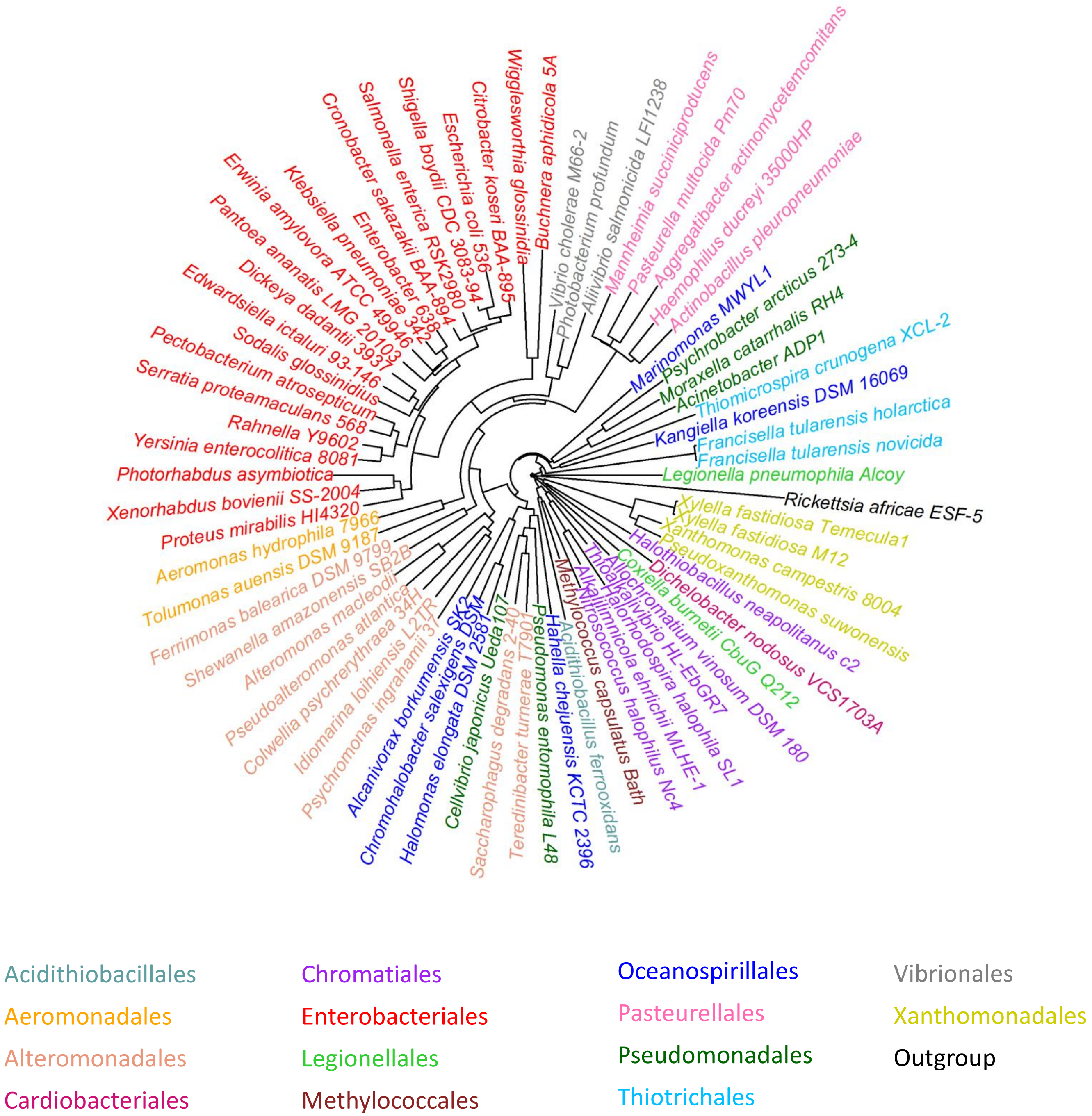

Buchnera aphidicola 5A
Wigglesworthia glossinidia
Citrobacter koseri BAA-895
Escherichia coli 536
Shigella boydii CDC 3083-94
Salmonella enterica RSK2980
Cronobacter sakazakii BAA-894
Enterobacter 638
Klebsiella pneumoniae 342
Erwinia amylovora ATCC 49946
Pantoea ananatis LMG 20103
Dickeya dadantii 3937
Edwardsiella ictaluri 93-146
Sodalis glossinidius
Pectobacterium atrosepticum
Serratia proteamaculans 568
Rahnella Y9602
Yersinia enterocolitica 8081
Photorhabdus asymbiotica
Xenorhabdus bovienii SS-2004
Proteus mirabilis HI4320
Aeromonas hydrophila 7966
Tolumonas auensis DSM 9187
Ferrimonas balearica DSM 9799
Shewanella amazonensis SB2B
Alteromonas macleodii
Pseudoalteromonas atlantica
Colwellia psychrerythraea 34H
Idiomarina loihiensis L2TR
Psychromonas ingrahamii 37
Alcanivorax borkumensis SK2
Chromohalobacter salexigens DSM
Halomonas elongata DSM 2581
Cellvibrio japonicus Ueda107
Saccharophagus degradans 2-40
Teredinibacter turnerae T7901
Pseudomonas entomophila L48
Hahella chejuensis KCTC 2396
Acidithiobacillus ferrooxidans
Methylococcus capsulatus Bath
Alkalilimnicola ehrlichii MLHE-1
Nitrosococcus halophilus Nc4
Halorhodospira halophila SL1
Thioalkalivibrio HL-EbGR7
Allochromatium vinosum DSM 180
Coxiella burnetii CbuG Q212
Dichelobacter nodosus VCS1703A
Halothiobacillus neapolitanus c2
Pseudoxanthomonas suwonensis
Xanthomonas campestris 8004
Xylella fastidiosa M12
Xylella fastidiosa Temecula1
Rickettsia africae ESF-5
Legionella pneumophila Alcoy
Francisella tularensis novicida
Francisella tularensis holarctica
Kangiella koreensis DSM 16069
Thiomicrospira crunogena XCL-2
Acinetobacter ADP1
Moraxella catarrhalis RH4
Psychrobacter arcticus 273-4
Marinomonas MWYL1
Actinobacillus pleuropneumoniae
Haemophilus ducreyi 35000HP
Aggregatibacter actinomycetemcomitans
Pasteurella multocida Pm70
Mannheimia succiniciproducens
Aliivibrio salmonicida LFI1238
Photobacterium profundum
Vibrio cholerae M66-2
Acidithiobacillales
Aeromonadales
Alteromonadales
Cardiobacteriales
Chromatiales
Enterobacteriales
Legionellales
Methylococcales
Oceanospirillales
Pasteurellales
Pseudomonadales
Thiotrichales
Vibrionales
Xanthomonadales
Outgroup

Figure S4

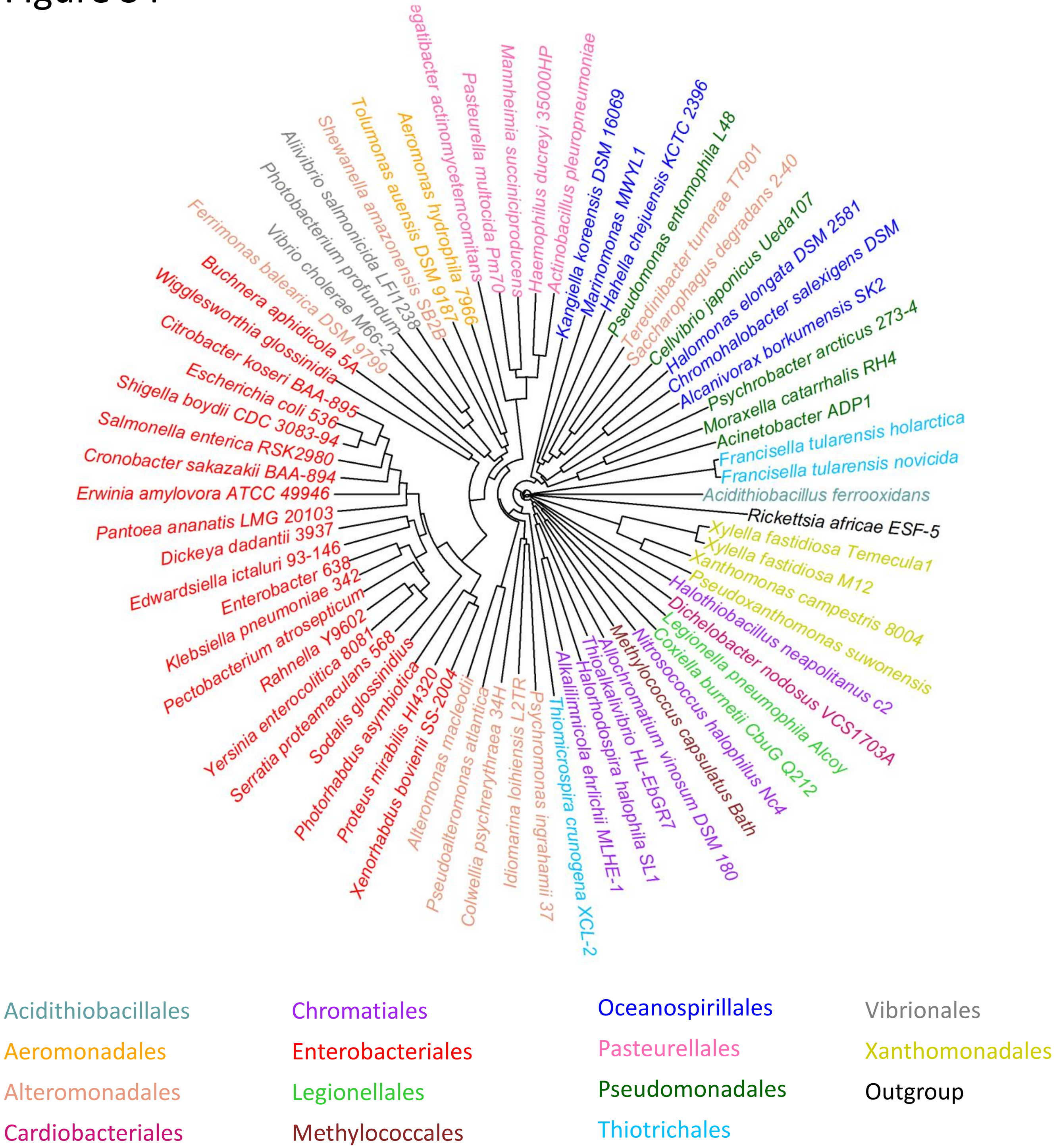
Acidithiobacillus ferrooxidans
Rickettsia africae ESF-5
Xylella fastidiosa Temecula1
Xylella fastidiosa M12
Xanthomonas campestris 8004
Pseudoxanthomonas suwonensis
Halothiobacillus neapolitanus c2
Dichelobacter nodosus VCS1703A
Legionella pneumophila Alcoy
Coxiella burnetii CbuG Q212
Nitrosococcus halophilus Nc4
Methylococcus capsulatus Bath
Allochromatium vinosum DSM 180
Thioalkalivibrio HL-EbGR7
Halorhodospira halophila SL1
Alkalilimnicola ehrlichii MLHE-1
Thiomicrospira crunogena XCL-2
Psychromonas ingrahamii 37
Idiomarina loihiensis L2TR
Colwellia psychrerythraea 34H
Pseudoalteromonas atlantica
Alteromonas macleodii
Xenorhabdus bovienii SS-2004
Proteus mirabilis HI4320
Photorhabdus asymbiotica
Sodalis glossinidius
Serratia proteamaculans 568
Yersinia enterocolitica 8081
Rahnella Y9602
Pectobacterium atrosepticum
Klebsiella pneumoniae 342
Enterobacter 638
Edwardsiella ictaluri 93-146
Dickeya dadantii 3937
Pantoea ananatis LMG 20103
Erwinia amylovora ATCC 49946
Cronobacter sakazakii BAA-894
Salmonella enterica RSK2980
Shigella boydii CDC 3083-94
Escherichia coli 536
Citrobacter koseri BAA-895
Wigglesworthia glossinidia
Buchnera aphidicola 5A
Ferrimonas balearica DSM 9799
Vibrio cholerae M66-2
Photobacterium profundum
Aliivibrio salmonicida LFI1238
Shewanella amazonensis SB2B
Tolumonas auensis DSM 9187
Aeromonas hydrophila 7966
egatibacter actinomycetemcomitans
Pasteurella multocida Pm70
Mannheimia succiniciproducens
Haemophilus ducreyi 35000HP
Actinobacillus pleuropneumoniae
Kangiella koreensis DSM 16069
Marinomonas MWYL1
Hahella chejuensis KCTC 2396
Pseudomonas entomophila L48
Teredinibacter turnerae T7901
Saccharophagus degradans 2-40
Cellvibrio japonicus Ueda107
Halomonas elongata DSM 2581
Chromohalobacter salexigens DSM
Alcanivorax borkumensis SK2
Psychrobacter arcticus 273-4
Moraxella catarrhalis RH4
Acinetobacter ADP1
Francisella tularensis holarctica
Francisella tularensis novicida
Acidithiobacillales
Aeromonadales
Alteromonadales
Cardiobacteriales
Chromatiales
Enterobacteriales
Legionellales
Methylococcales
Oceanospirillales
Pasteurellales
Pseudomonadales
Thiotrichales
Vibrionales
Xanthomonadales
Outgroup

Figure S5

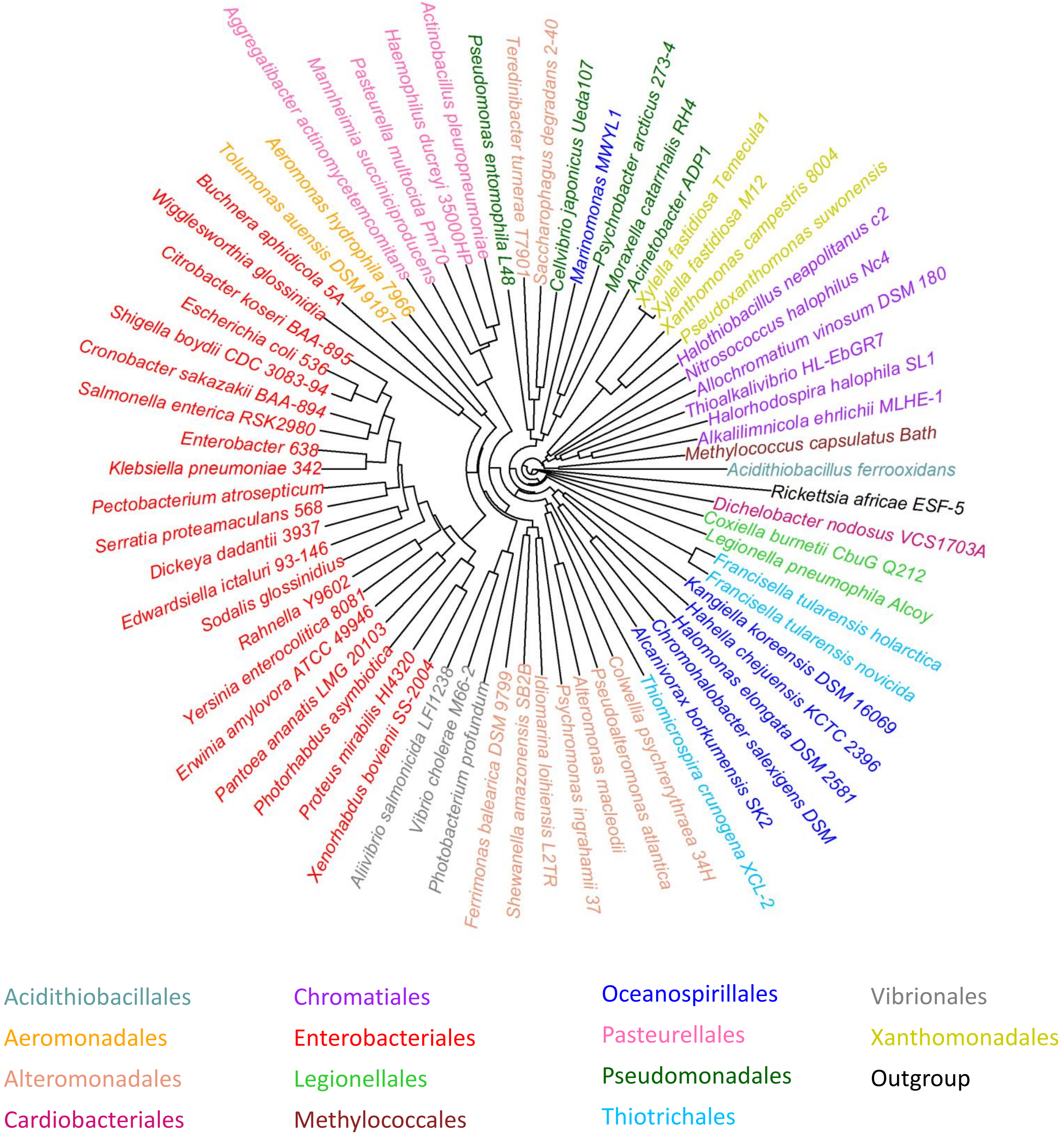
Aggregatibacter actinomycetemcomitans
Mannheimia succiniciproducens
Pasteurella multocida Pm70
Haemophilus ducreyi 35000HP
Actinobacillus pleuropneumoniae
Pseudomonas entomophila L48
Teredinibacter turnerae T7901
Saccharophagus degradans 2-40
Cellvibrio japonicus Ueda107
Marinomonas MWYL1
Psychrobacter arcticus 273-4
Moraxella catarrhalis RH4
Acinetobacter ADP1
Xylella fastidiosa Temecula1
Xylella fastidiosa M12
Xanthomonas campestris 8004
Pseudoxanthomonas suwonensis
Halothiobacillus neapolitanus c2
Nitrosococcus halophilus Nc4
Allochromatium vinosum DSM 180
Thioalkalivibrio HL-EbGR7
Halorhodospira halophila SL1
Alkalilimnicola ehrlichii MLHE-1
Methylococcus capsulatus Bath
Acidithiobacillus ferrooxidans
Rickettsia africae ESF-5
Dichelobacter nodosus VCS1703A
Coxiella burnetii CbuG Q212
Legionella pneumophila Alcoy
Francisella tularensis holarctica
Francisella tularensis novicida
Kangiella koreensis DSM 16069
Hahella chejuensis KCTC 2396
Halomonas elongata DSM 2581
Chromohalobacter salexigens DSM
Alcanivorax borkumensis SK2
Thiomicrospira crunogena XCL-2
Colwellia psychrerythraea 34H
Pseudoalteromonas atlantica
Alteromonas macleodii
Psychromonas ingrahamii 37
Idiomarina loihiensis L2TR
Shewanella amazonensis SB2B
Ferrimonas balearica DSM 9799
Photobacterium profundum
Vibrio cholerae M66-2
Aliivibrio salmonicida LFI1238
Xenorhabdus bovienii SS-2004
Proteus mirabilis HI4320
Photorhabdus asymbiotica
Pantoea ananatis LMG 20103
Erwinia amylovora ATCC 49946
Yersinia enterocolitica 8081
Rahnella Y9602
Sodalis glossinidius
Edwardsiella ictaluri 93-146
Dickeya dadantii 3937
Serratia proteamaculans 568
Pectobacterium atrosepticum
Klebsiella pneumoniae 342
Enterobacter 638
Salmonella enterica RSK2980
Cronobacter sakazakii BAA-894
Shigella boydii CDC 3083-94
Escherichia coli 536
Citrobacter koseri BAA-895
Wigglesworthia glossinidia
Buchnera aphidicola 5A
Tolumonas auensis DSM 9187
Aeromonas hydrophila 7966
Acidithiobacillales
Chromatiales
Oceanospirillales
Vibrionales
Aeromonadales
Enterobacteriales
Pasteurellales
Xanthomonadales
Alteromonadales
Legionellales
Pseudomonadales
Outgroup
Cardiobacteriales
Methylococcales
Thiotrichales

Figure S6

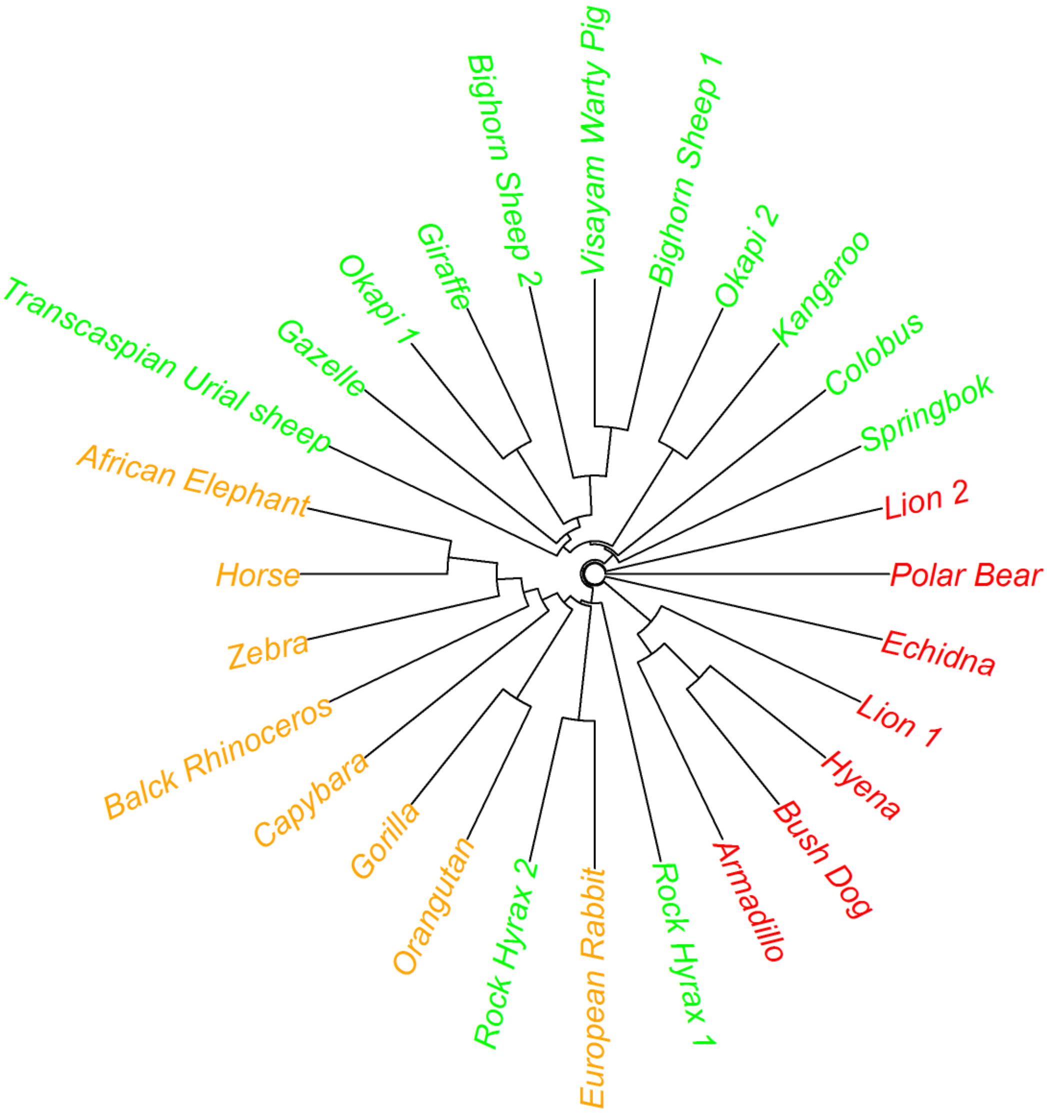

Herbivore Foregut **Herbivore Hindgut** **Carnivore**

Figure S7

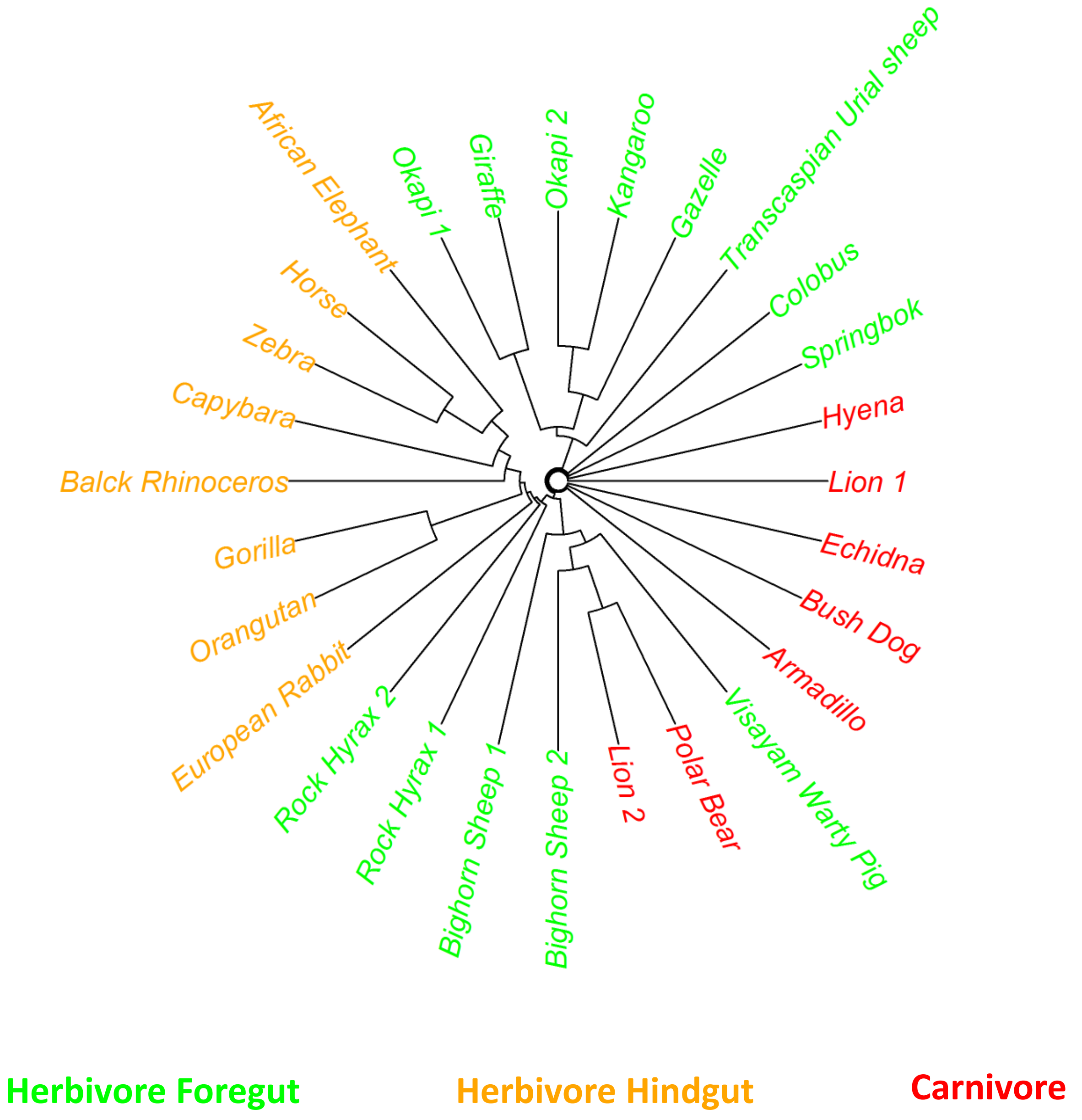

African Elephant
Okapi 1
Giraffe
Okapi 2
Kangaroo
Gazelle
Transcaspian Urial sheep
Colobus
Springbok
Hyena
Lion 1
Echidna
Bush Dog
Armadillo
Visayam Warty Pig
Polar Bear
Lion 2
Bighorn Sheep 2
Bighorn Sheep 1
Rock Hyrax 1
Rock Hyrax 2
European Rabbit
Orangutan
Gorilla
Balck Rhinoceros
Capybara
Zebra
Horse
Herbivore Foregut
Herbivore Hindgut
Carnivore

Figure S8

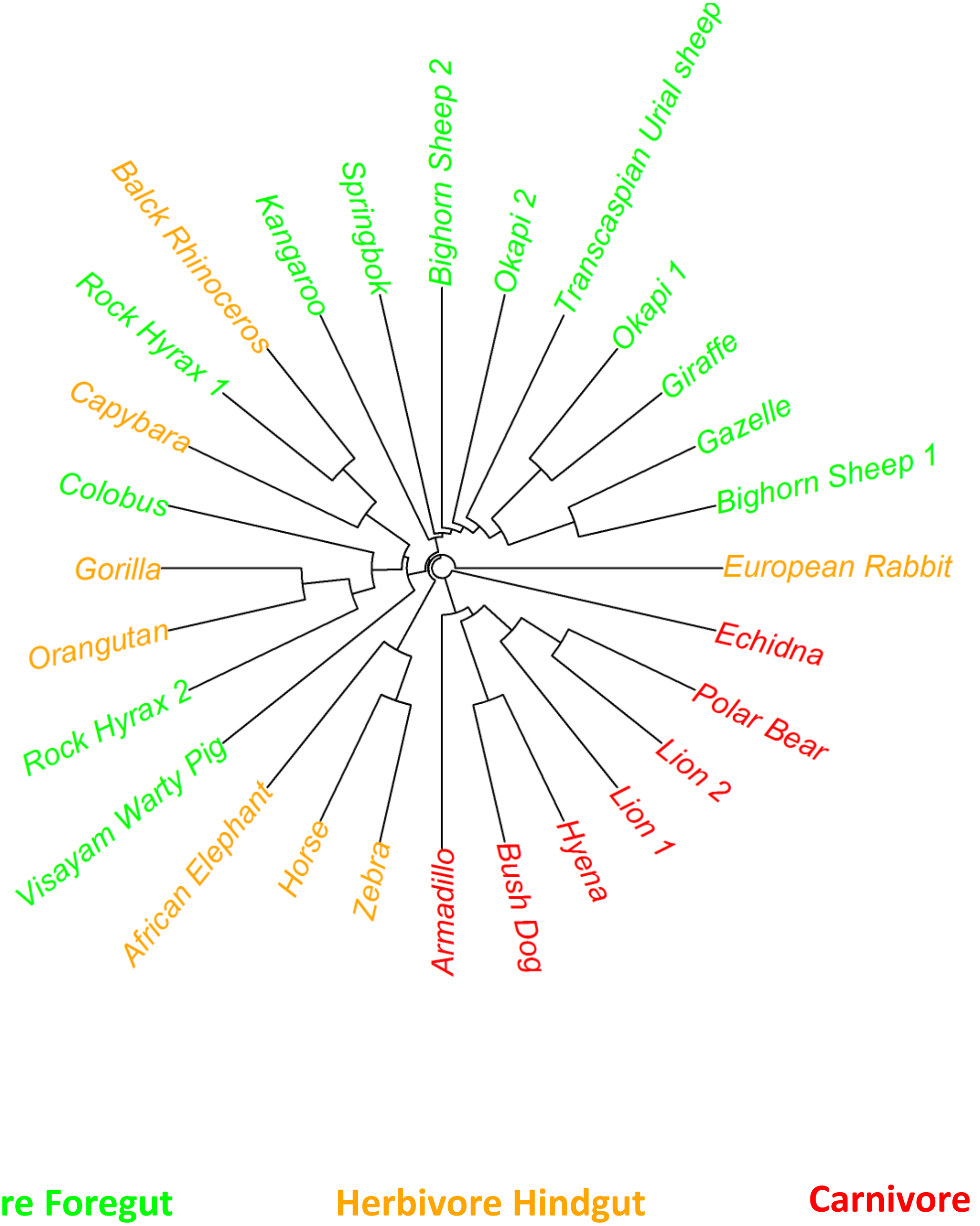

Bighorn Sheep 2
Transcaspian Urial sheep
Springbok
Okapi 2
Kangaroo
Balck Rhinoceros
Okapi 1
Giraffe
Rock Hyrax 1
Gazelle
Capybara
Bighorn Sheep 1
Colobus
Gorilla
European Rabbit
Orangutan
Echidna
Rock Hyrax 2
Polar Bear
Visayam Warty Pig
Lion 2
African Elephant
Lion 1
Horse
Hyena
Zebra
Armadillo
Bush Dog
Herbivore Foregut
Herbivore Hindgut
Carnivore
